# Integrating multi‐omics features enables non‐invasive early diagnosis and treatment response prediction of diffuse large B‐cell lymphoma

**DOI:** 10.1002/ctm2.70174

**Published:** 2025-01-07

**Authors:** Weilong Zhang, Bangquan Ye, Yang Song, Ping Yang, Wenzhe Si, Hairong Jing, Fan Yang, Dan Yuan, Zhihong Wu, Jiahao Lyu, Kang Peng, Xu Zhang, Lingli Wang, Yan Li, Yan Liu, Chaoling Wu, Xiaoyu Hao, Yuqi Zhang, Wenxin Qi, Jing Wang, Fei Dong, Zijian Zhao, Hongmei Jing, Yanzhao Li

**Affiliations:** ^1^ Department of Hematology Lymphoma Research Center Peking University Third Hospital Beijing China; ^2^ BOE Technology Group Co., Ltd Beijing China; ^3^ Department of Laboratory Medicine Peking University Third Hospital Beijing China

**Keywords:** cfDNA, DLBCL, early diagnosis, integrated model, multi‐omics, treatment prediction

## Abstract

**Background:**

Multi‐omics features of cell‐free DNA (cfDNA) can effectively improve the performance of non‐invasive early diagnosis and prognosis of cancer. However, multimodal characterization of cfDNA remains technically challenging.

**Methods:**

We developed a comprehensive multi‐omics solution (COMOS) to specifically obtain an extensive fragmentomics landscape, presented by breakpoint characteristics of nucleosomes, CpG islands, DNase clusters and enhancers, besides typical methylation, copy number alteration of cfDNA. The COMOS was tested on 214 plasma samples of diffuse large B‐cell lymphoma (DLBCL) and matched healthy controls.

**Results:**

For early diagnosis, COMOS improved the area under the curve (AUC) value to .993 compared with the individual omics model, with a sensitivity of 95% at 98% specificity. Detection sensitivity achieved 91% at 99% specificity in early‐stage patients, while the AUC values of the individual omics model were 0.942, 0.968, 0.989, 0.935, 0.921, 0.781 and 0.917, respectively, with lower sensitivity and specificity. In the treatment response cohort, COMOS yielded a superior sensitivity of 88% at 86% specificity (AUC, 0.903). COMOS has achieved excellent performance in early diagnosis and treatment response prediction.

**Conclusions:**

Our study provides an effectively improved approach with high accuracy for the diagnosis and prognosis of DLBCL, showing great potential for future clinical application.

**Key points:**

- A comprehensive multi‐omics solution to specifically obtain an extensive fragmentomics landscape, presented by breakpoint characteristics of nucleosomes, CpG islands, DNase clusters and enhancers, besides typical methylation, copy number alteration of cfDNA.
- Integrated model of cfDNA multi‐omics could be used for non‐invasive early diagnosis of DLBCL.
- Integrated model of cfDNA multi‐omics could effectively evaluate the efficacy of R‐CHOP before DLBCL treatment.

## Graphical abstract

A comprehensive multi‐omics solution capable of obtaining fragmentomics landscape of cfDNA was developed and followed by an integrated model for non‐invasive early diagnosis of DLBCL, achieving excellent efficacy of R‐CHOP before DLBCL treatment.

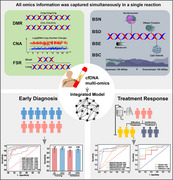

## 1 BACKGROUND

Diffuse large B‐cell lymphoma (DLBCL) is an aggressive form of mature B cells and is the most common type of non‐Hodgkin lymphoma. About 30% of DLBCL patients present with localized stage disease, which can also be defined as stage I or stage II, and the rest 70% present with late stage.^1^, ^2^ Clinical studies show that the overall survival (OS) rate of early‐stage DLBCL is 95% at 2 years and 92% at 4 years,^3^ while for advanced DLBCL, the OS rate ranges from 67.4% for stage I disease to 55.2% for stage IV disease.^4^ On the other hand, Rituximab plus cyclophosphamide, doxorubicin, vincristine and prednisone (R‐CHOP) is considered as the gold standard for first‐line treatment,^5^, ^6^, ^7^ but still 30−50% of patients are ineffective in treatment, with a significantly poor prognosis and a median OS of 6.3 months.^8^ Therefore, early diagnosis and effective treatment prediction of DLBCL can significantly improve the OS and prognosis.

However, there is currently a lack of adequate technical tools for early diagnosis and treatment response prediction of DLBCL. Traditional diagnostic methods, such as positron emission tomography/computed tomography (PET/CT) and the international prognostic index (IPI) for DLBCL, rely on detailed examination of tumour tissue, but leading to sampling errors or false negative results because of tumour heterogeneity and concomitant inflammation.^9^, ^10^, ^11^, ^12^ Meanwhile, protein markers including CD5,^13^, ^14^ CD30^15^, ^16^ and Ki67^17^, ^18^ have been developed to predict treatment response in DLBCL patients, although the results are often controversial. Molecular characteristics such as gene mutation, abnormal methylation patterns, genomic copy number alteration (CNA) and fragmentation features carried by cell‐free DNA (cfDNA) have been extensively used in cancer early diagnosis, treatment response prediction, prognosis monitoring and other scenarios.^19^, ^20^, ^21^, ^22^, ^23^, ^24^, ^25^ However, individual omics analysis of cfDNA only exhibits limited detection capabilities, especially for stage I cancer cases.^26^, ^27^, ^28^, ^29^ Multi‐omics research has been emerging in recent years as a highly sensitive early diagnosis method.^30^, ^31^ Integration of cfDNA epigenomics and fragmentomics has been proven to improve the diagnostic performance of lung cancer patients,^32^ and the inclusion of CNA data could further increase the sensitivity and specificity by constructing an ensemble model.^33^, ^34^ Although some progress has been made, the existing techniques mainly use low‐depth whole‐genome sequencing, which only offers basic methylation, CNA and fragmentation, but cannot provide large‐scale omics information across different resolutions, especially for cfDNA fragmentation patterns. Previous studies demonstrate that DNase I hypersensitive Clusters (DNase Clusters), located in chromatin open regions, could infer the tissue origin of plasma DNA to predict the tumour location in cancer patients.^35^, ^36^, ^37^ In addition, the CpG islands, promoters and enhancers contained in this region are related to tumour formation.^38^ However, those regions usually cannot be effectively sequenced because they are shelterless and easily degraded. Nucleosomes in closed chromatin regions form fragmentation patterns that can be used for tissue tracing and cancer diagnosis.^39^ Therefore, an effective approach that can provide all of these chromatin‐related features is essential for both the diagnosis and treatment prediction of DLBCL.

Herein, to obtain a complete informatics of the cfDNA, we showcased a comprehensive multi‐omics solutions (COMOS) integrating breakpoint scores for regions surrounding nucleosomes (BSN), CpG islands (BSC), DNase clusters (BSD) and enhancers (BSE) with differential methylated regions (DMRs), fragment size ratio (FSR) and CNA. Further, 214 clinical cohort samples, including 117 healthy controls and 97 DLBCL patients, were tested for clinical verification and demonstrated an outstanding performance for early diagnosis and treatment response prediction.

## 2 METHODS

### 2.1 Patients and sample characteristics

This study included 119 DLBCL patient samples and 117 healthy control samples from Peking University Third Hospital (Table S1). In all cases, DLBCL was diagnosed using appropriate diagnostic criteria from the 2016 WHO classification of lymphoid neoplasms, and 81 patients received standard R‐CHOP therapy. Before any treatment, lactate dehydrogenase (LDH), beta2 microglobulin (β2MG) and cfDNA were collected. Disease stage was defined by the Ann Arbor staging system, this study was conducted in accordance with the Declaration of Helsinki, and all samples had signed patient consent. In the actual data analysis, due to the small amount of DMRs in some patient samples, 97 patients among 119 patients were retained for early diagnosis cohort study, and 80 patients among 81 follow‐up samples treated with R‐CHOP were retained for further treatment response cohort study. Some of the discarded samples participated in the DMR identification process.

### 2.2 Isolation of plasma cfDNA

In this study, 2–8 mL of whole blood was collected into 8.5 mL Cell‐Free DNA Blood Collection Tubes (Cat#07785674001, Roche), and plasma separation was performed within 72 h. First, we centrifuged at 1350 × *g* for 12 min at 4°C, carefully removed the light yellow supernatant liquid, to avoid contaminating the white blood cell layer, and immediately transferred the supernatant liquid to a 2 mL DNase‐free sterile centrifuge tube. Second, we centrifuged at 13 500 × *g* for 5 min at 4°C, transferred the supernatant to a 2 mL DNase‐free sterile centrifuge tube, and then used QIAamp MinElute ccfDNA Mini Kit (Cat#55204, Qiagen) to extract cfDNA, followed by using Qubit dsDNA HS Assay Kit (Cat#Q32854, Thermo Fisher Scientific) to determine DNA concentration and using Agilent 4200 TapeStation (Agilent) for fragment analysis.

### 2.3 Library preparation

We constructed methylation libraries by combining multiple kits, and used NadPrep Methyl Library Preparation Module (Cat#1002502, Nanodigmbio) to end repair cfDNA, add ‘A’, and ligase adapters containing fully cytosine‐methylated unique molecular identifier (UMI). For methylation conversion, NEBNEXT Enzymatic Methyl‐seq Conversion Module (Cat#E7125L, New England Biolabs) was used. The methylation library used the Qubit dsDNA HS Assay Kit (Cat#Q32854, Thermo Fisher Scientific) to determine the concentration and Agilent 4200 TapeStation for library fragment analysis.

### 2.4 Target region capture sequencing

Target region hybridization was performed by Twist Fast Hybridization and Wash Kit (Cat#101174, Twist Bioscience). We mixed eight methylation libraries with different indexes; each library was 187.5 ng. The resulting mixture was combined with 4 µL Twist Custom Methylation Panel (Cat#105520, Twist Bioscience), 8 µL Universal Blockers and 5 µL Blocker Solution (Cat#103557, Twist Bioscience) and concentrated to a dry powder state. The Custom Methylation Panel covered 123 M methylation region, which came from the latest database versions UCSC, Ensemble, ENCODE and so on.Thereafter, the powder was dissolved by 20 µL Fast Hybridization mix, then 30 µL Enhancer was added and mixed thoroughly. The following condition was carried out: incubation at 95°C for 5 min, then 60°C for 16–24 h. After hybridization was completed, streptavidin beads were used for binding and cleaning. The product was amplified and enriched by 25 µL KAPA HiFi Hotstart Readymix (Cat#07958927001, Roche) and 2.5 µL Amplification Primer under the following conditions: pre‐denaturation at 98°C for 45 s, denaturation at 98°C for 15 s, annealing at 60°C for 30 s, extension at 72°C for 30 s, including denaturation to extension steps for eight cycles, and final extension at 72°C for 1 min. The sequencing platform is Illumina NovaSeq 6000 (Illumina), and the sequencing raw data for each sample was approximately 20–40G.

### 2.5 Sequencing data processing and comparison

We used umitools^40^ (v1.1.2) to extract the UMI of each read and merged it into the fastq sequence identifier, then used trim_galore (v0.6.6) to filter the data, and used bismark^41^ (v0.23.1) to map the filtered fastq to the hg19 reference genome prepared through genome preparation, and the ‘deduplicate_bismark –barcode’ mode was used to deduplicate.

### 2.6 Multimodal information extraction

#### 2.6.1 Methylation signal

The methylation information was extracted from the deduplicated alignment file, and the methylated C and unmethylated C of each CpG site were obtained in the customized Panel interval number of bases to facilitate subsequent analysis. The detection of DMR was completed using metilene^42^ (v0.2). The minimum number of CpGs covered by each DMR region was set to 4, and the difference significance *q*‐value and average methylation difference were set to 0.05 and 20 (NC vs. T)/15 (PRCR vs. PDSD). We calculated the methylation level of the sample on each DMR obtained, and counted the DMR missing proportion of each sample. When the missing proportion was greater than 20%, the sample was excluded (including training and validation samples of the set) and did not participate in subsequent modelling steps. The missing values of the remaining samples were filled with the mean methylation rate of the DMR itself. The identification of DMR was only performed on the training set.

#### 2.6.2 Short fragment ratio

The autosomal region of the genome was divided into 5‐Mb intervals, and a total of 589 bins were obtained. We used the in‐house script to count the start, end and length of the fragments of the comparison files after deduplication, and used bedtools intersect^43^ (v2.26.0) to obtain the intersection area based on the 589 bins, by setting the ‘−F’ parameter to 0.5, only reads with fragment lengths ≥100 and ≤220 were extracted, and finally 567 bins are reserved. Fragments with lengths between 100 and 150 were defined as short fragments, while fragments with lengths between 151 and 220 were defined as long fragments, and the ratio of the number of short fragments to the number of long fragments was calculated as the short fragment ratio.

#### 2.6.3 Copy number variation

We used QDNAseq^44^ (v1.34.0) to read the alignment file after deduplicated and sorted, set the bin size to 1000 Kbp, used ‘applyFilters’ to filter the bin interval, and used ‘estimateCorrection’ and ‘correctBins’ to estimate correction to read counts and correct binned read counts for GC content and mappability, used ‘normalizeBins’ and ‘smoothOutlierBins’ to obtain the final CNA data. After the above steps, each sample obtained statistical results of 1942 bins.

#### 2.6.4 Breakpoint score of chromatin‐related features

We obtained the whole‐genome nucleosome map CA01 file from a previous study,^39^ and downloaded a total of three chromatin‐related region files including CpG islands, DNase Clustered and Enhancers from the UCSC website. We calculated the distance between the breakage start site of each fragment and the centre point of all chromatin‐related regions, and counted the proportion of broken fragments within the range of 200, 300, 150 and 500 bp on the upstream and downstream of the centre point of the above chromatin‐related.

### 2.7 Analysis of methylation levels in standard samples

We extracted CpG site methylation levels and performed depth statistics on gradient methylation level standard samples with different DNA contents. The methylation levels of CpG sites on different gradient methylation level standard samples with the same DNA content were fit by using the linear regression function of R and *p*‐values were calculated.

### 2.8 Model construction

We defined three classifiers, respectively: logistic regression, random forest and AdaBoost, and defined random seeds as 42. Standardization was performed using the sklearn StandardScaler function. Specifically, we used StandardScaler to fit the training set and normalized the entire dataset. For each omics data, the model construction methods of different classifiers were as follows:

In the training set, recursive feature elimination with cross‐validation (RFECV) was used for feature screening, where cv was set to 10 and the evaluation metric was set to ‘roc_auc’.

We utilized ‘GridSearchCV’ for hyperparameter tuning on the training set data after feature selection, set cv to 10 and evaluation metric to ‘roc_auc’. The specific hyperparameter list is as follows:

AdaBoost: ‘n_estimators’: [50, 100, 200], ‘learning_rate’: [0.01, 0.1, 1.0]
LogisticRegression: ‘C’: [0.001, 0.01, 0.1, 1, 10, 100], ‘penalty’: [‘l1’, ‘l2’], ‘max_iter’: [100, 500, 1000]
RandomForest: ‘n_estimators’: [100, 200, 300], ‘max_depth’: [None, 10, 20, 30], ‘min_samples_split’: [2, 5, 10], ‘min_samples_leaf’: [1, 2, 4], ‘max_features’: [‘auto’, ‘sqrt’, ‘log2’]

We performed 10‐fold cross‐validation on each classifier after the hyperparameters, and calculated the average area under the curve (AUC).

For each classifier after the hyperparameters, we fit it on the training set, and performed prediction evaluation on the verification set, and computed the performance metrics related to the model.

### 2.9 Model integration

By computing the average of 10‐fold AUC of three classifiers after the hyperparameters under each omics, the model with the largest average of AUC was the optimal model for the omics. We set the initial classification threshold to 0.5, increased it in steps of 0.01, counted the sensitivity of the optimal model under each omics when the specificity reaches 99% on the training set and saved the current classification threshold for subsequent analysis. We expected that the classification threshold obtained through this step could make the specificity of the model in the validation set high enough, in order to meet the high requirements of specificity in different tasks (early diagnosis or treatment response prediction). The positive prediction probability value generated by seven individual omics optimal classification model was used as the feature of integrated model training. Specifically, we concatenated the positive prediction probabilities of the seven individual omics as columns, with each row representing the positive prediction probabilities for a sample across different omics. The sample cohorts for the training and test sets were the same as those for the single‐omics datasets. We used the random forest to build the COMOS model on the dataset of all individual omics positive predictive probability, and the random seed was set to 42. The default parameters were used for early diagnosis, and since the sample size of the treatment response prediction is smaller than that of the early diagnosis, the n_estimators was set to 5 to prevent the model from over‐fitting and reduce the model complexity. Similarly, the classification threshold when the specificity of the integrated model reaches 99% on the training set was saved for subsequent analysis.

### 2.10 Model evaluation

The 1000 bootstrap resamples were used to obtain the 95% confidence intervals of AUC, sensitivity, specificity and other indicators. The DelongTest method was used to calculate the difference between the two models.

In the early diagnosis of cancer at different stages, due to the limited number of patients, cancer stages I and II were combined into the early stage, and cancer stages III and IV were combined into the late stage. We utilized the Wilson method to separately assess the diagnostic ability of the model in early‐ or late‐stage patients and normal control sample sets, and calculated the 95% confidence intervals.

### 2.11 Statistical analysis

The heatmap for early diagnosis was drawn by calculating the Z‐score for each omics feature in tumour patients relative to normal controls. The calculation method for the Z‐score of a cancer group/normal control group was: $Z_{T}=\frac{X_{T}-\mu_{\mathrm{NC}}}{\sigma_{\mathrm{NC}}}/Z_{\mathrm{NC}}=\frac{X_{\mathrm{NC}}-\mu_{\mathrm{NC}}}{\sigma_{\mathrm{NC}}}$, and mapped the Z‐score value to the corresponding colour. t‐SNE dimensionality reduction was accomplished using the t‐Distributed Stochastic Neighbour Embedding (tSNE) function from the sklearn (0.24.2) package,^45^ and ‘n_components’ and the random seed was set to 2 and 42. We used ChIPseeker^46^ (v1.34.1) to annotate DMRs to related genes and genomic elements, set the TSS region range to −2 Kbp to 2 Kbp and used clusterProfiler^47^ (v4.6.2) to annotate related KEGG signalling pathways. In order to compare the advantages and disadvantages of the treatment response prediction model and the clinical markers LDH and β2MG, the AUC of LDH and β2MG under a certain number of samples were calculated by numerical integration method. This study used the sklearn package of Python (v3.6.9) for machine learning modelling, and matplotlib and R for plotting.

## 3 RESULTS

### 3.1 COMOS overview for methylation, CNA and fragmentation detection

**FIGURE 1:**
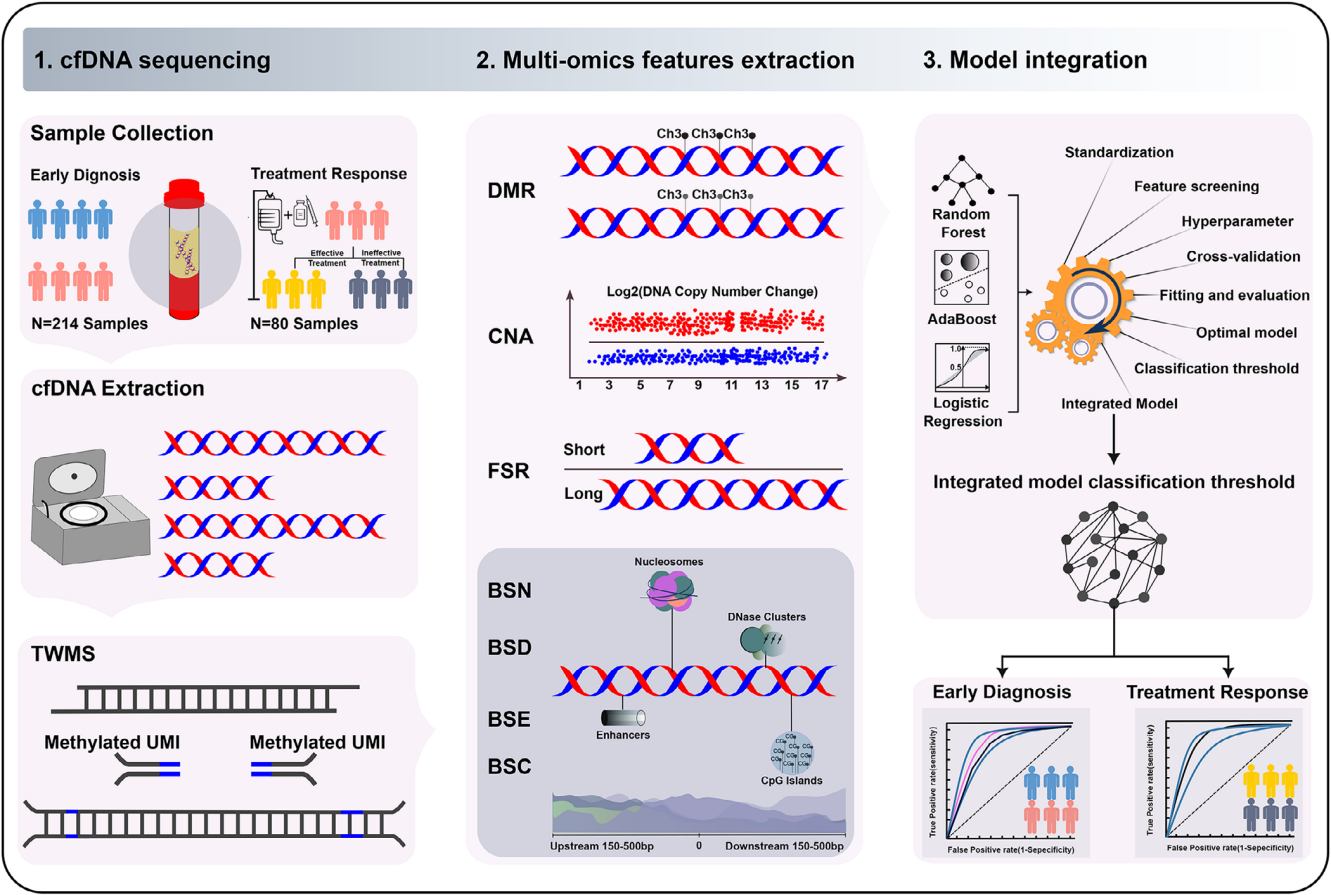
Workflow of the whole experiment and model construction. Plasma samples were collected from healthy controls and patients, respectively. Then, cfDNA was extracted and subjected to TWMS (target whole methylome sequencing), and omics data of DMR, CNA, FSR, BSN, BSC, BSD and BSE were analysed. Individual omics optimal model was obtained through three algorithm models of random forest, AdaBoost, logistic regression algorithms, followed by model training to obtain seven optimal individual omics models, and the positive prediction probability of these models on the training set were used for integration to construct the integrated model. Validation sets were used for evaluation.

The COMOS approach mainly included three steps, a deep targeted whole methylome sequencing (TWMS), multi‐omics features extraction and model integration (Figure 1). To preserve the integrity of the cfDNA,^48^ an enzyme‐mediated methylation conversion was utilized to construct a sequencing library containing a fully cytosine‐methylated UMI. The whole target methylome was then captured by hybridization to a custom probe panel (Figure S1A,B). CNA, FSR, BSN, BSC, BSD and BSE were consequently extracted and utilized to construct an integrating model for early diagnosis and treatment response prediction of DLBCL.

**FIGURE 2:**
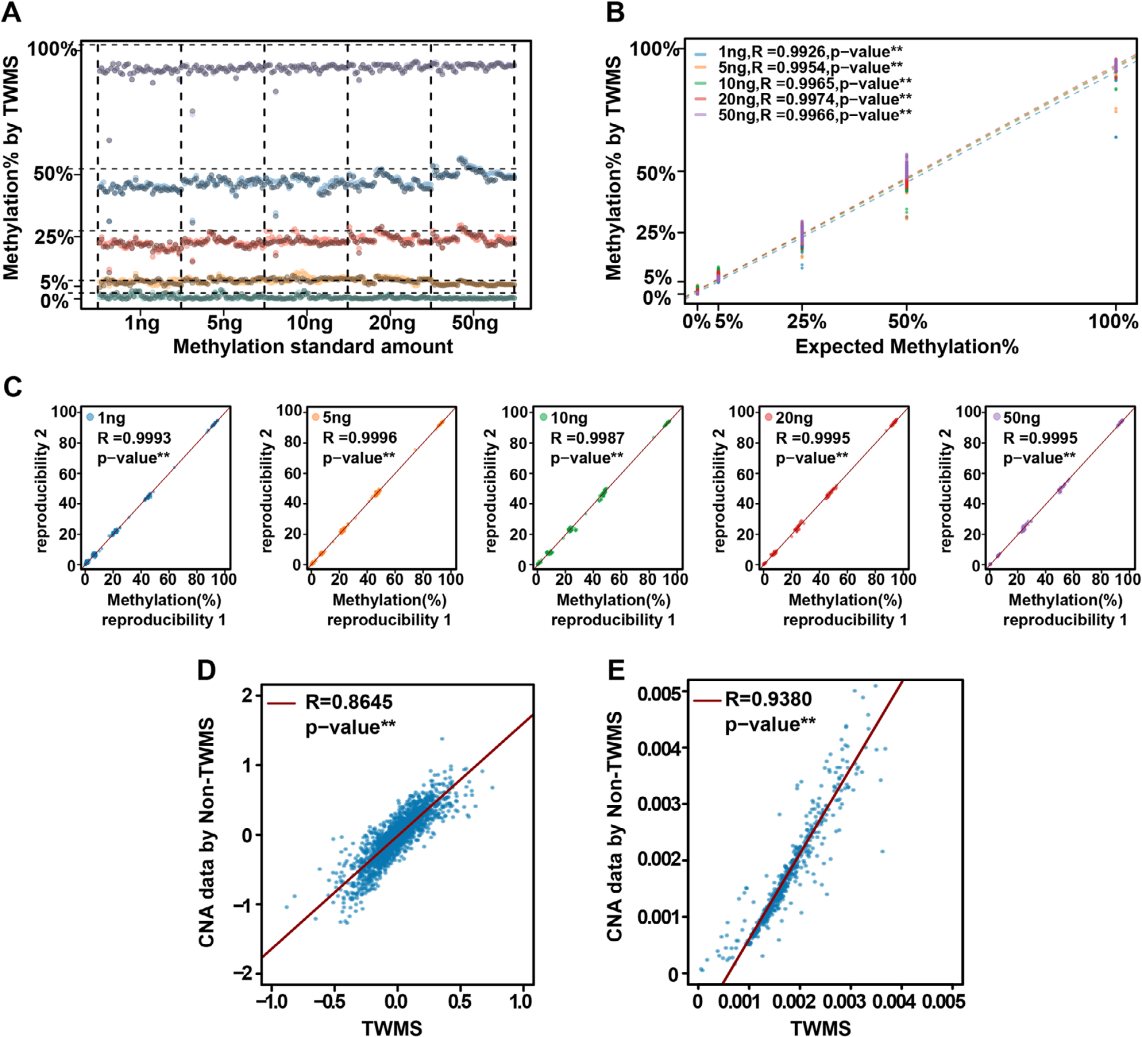
Analysis of TWMS performance in the methylation, CNA and fragmentation. (A) The amount of input was 1, 5, 10, 20 and 50 ng, respectively, and the methylation rate was 0%, 5%, 25%, 50% and 100%, respectively. (B) Consistency of the expected frequency with the observed amount detected by TWMS in the methylation detection assay. (C) Reproducibility of TWMS in analysing DNA methylation. Correlation of the methylation between replicates in each target is shown in the dot plot, *n* = 2 biological replicates. (D) Scatter plot between CNA profiles of an individual sample with the reference, based on TWMS and non‐TWMS data, respectively. The regression line is coloured in red. (E) Scatter plot from TWMS and non‐TWMS data of an individual sample. The regression line is coloured in red.

To verify methylation detection of TWMS, the fully methylated human genomic DNA and unmethylated DNA were mixed to prepare reference standards with 0%, 5%, 25%, 50% and 100% methylation frequencies. Standards of 1, 5, 10, 20 and 50 ng were input for each sequencing experiment with two technical replicates, and the methylation sites with coverage of ≥300 were selected for statistical analysis. The results showed that the methylation frequencies of 0−100% were detected, consistent with theoretical frequencies, and displayed a linear correlation (*R* = .99, *p* <0.01, Figure 2A,B). Furthermore, as little as 1 ng of sample could be detected and the detection capability was reproducible at all methylation sites evaluated (*R* = 0.99, *p* < 0.01, Figure 2C). These results confirmed the reliability of TWMS in detecting methylation variations.

Given that TWMS ensured the integrity of cfDNA and covered the entire methylation region, it was theoretically effective to obtain a large amount of CNA and fragmentation information. Therefore, to evaluate the feasibility of using the TWMS methylation data for fragmentation and CNA analysis, we randomly included four cancer patient samples to perform a consistency analysis between TWMS and non‐methylated converted TWMS (non‐TWMS) data (see Methods − Multimodal information extraction for details). TWMS and non‐TWMS data from sample 4–14 and other samples 4–06, 4–65 and 4–36 (Figure S2A−C) showed a highly consistent copy number pattern with a linear correlation (*R* = .86, *p* < .01, Figure 2D). Similarly, cfDNA fragmentation analysis of sample 4–14 and other cancer samples was performed simultaneously (Figure S2D−F). The ratio of the number of fragments in each 5‐Mb interval to the total number of fragments was calculated (see Methods − Multimodal information extraction for details), which showed a highly consistent fragmentation pattern and a linear correlation (*R* = .94, *p* < .01, Figure 2E). These results demonstrated the accuracy of TWMS in detecting CNA and fragmentation. Besides, a fully cytosine‐methylated UMI adapter (Figure S1B) was used to perform deduplication analysis, which increased the number of unique reads and avoided excessive deduplication (Figure S1C). In short, the COMOS approach, on the basis of an elaborate experimental design and data analysis, could improve the detection accuracy of methylation, CNA and fragmentation.

### 3.2 Multi‐omics profiling between DLBCL and healthy controls

**FIGURE 3:**
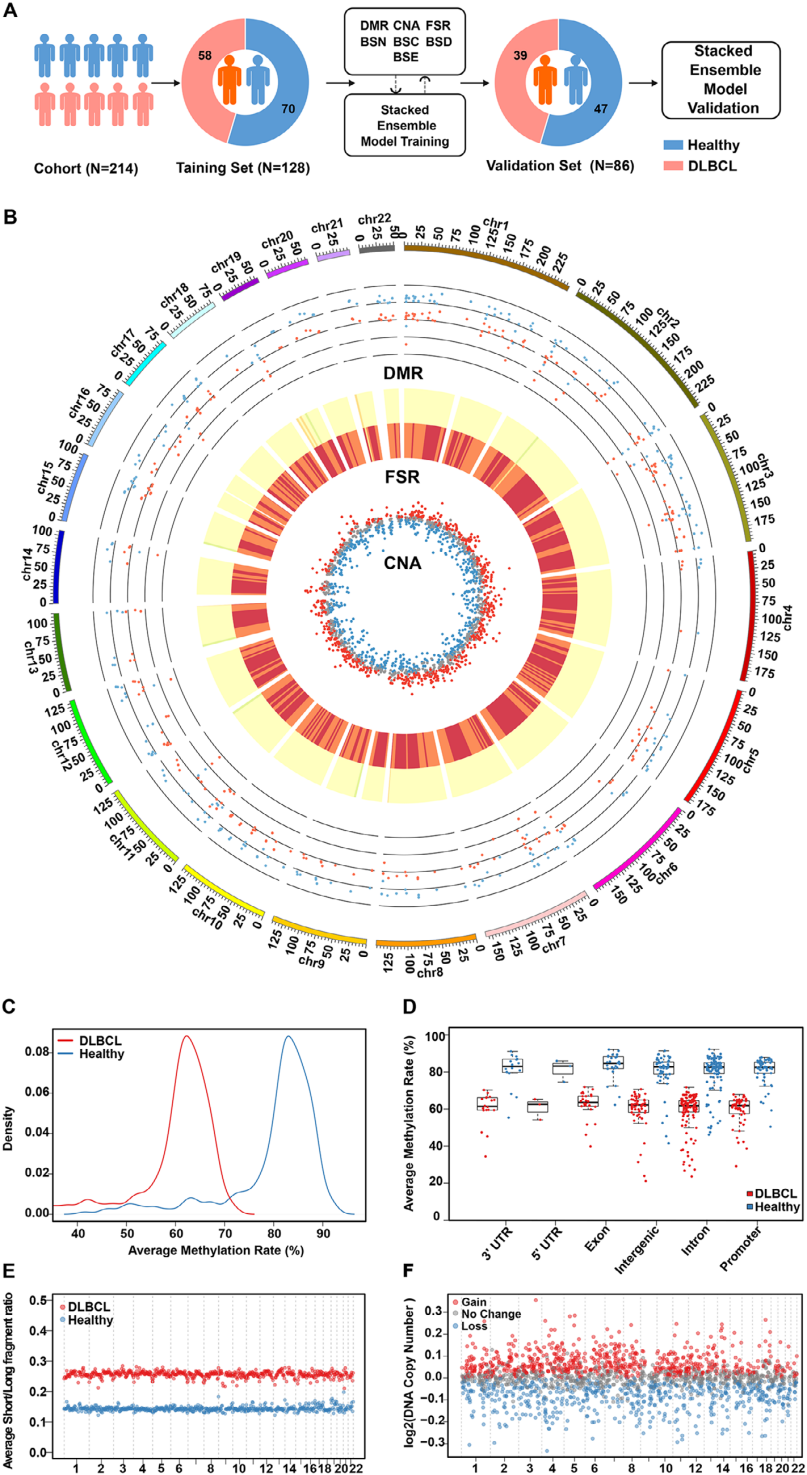
Difference characteristics analysis of methylation, CNA and fragmentation in early diagnosis cohort. (A) A total of 214 participants (DLBCL = 97, Healthy Control = 117) were included for model construction and validation. (B) Distribution of DMR, FSR and CNA on chromosomes between cancer group and normal control group. DMR: The average methylation rates of samples from different cohorts on DMR, red dots represent cancer group, blue dots represent normal control group; FSR: A heatmap illustrating the average FSR across different cohorts on all 5 Mb intervals, with the outer circle representing normal control group and the inner circle representing cancer group; CNA: Same as Figure 3F. (C) The distribution curves of methylation rate density in DMR. (D) Methylation ratio of the genomic element region of the cancer group and the normal control group. (E) The average FSR of the cancer group and the normal control group in each 5 Mb interval. (F) The copy number variations in the cancer group relative to the normal control group, that is log2 (T/NC), and perform the rank sum test. When *p*‐value < .05 and log2 (T/NC) is greater than 0 or less than 0, it is judged as Copy number Gain or Loss, otherwise, the copy number is regarded as No Change.

In order to comprehensively prove the performance of COMOS in cancer detection, a clinical cohort sample study was conducted and validated, including 117 healthy controls and 97 pathologically confirmed DLBCL patients (Tables S2). In the early diagnosis study, stages I and II were used as early stage, stages III and IV as late stage, and the cohort samples were divided proportionally into the training set (healthy control = 70, DLBCL = 58) and the verification set (healthy control = 47, DLBCL = 39). The extracted DMR, CNA, FSR, BSN, BSC, BSD and BSE features from the training set were used to build an integrating model, and then evaluated on the validation set (Figure 3A). Overall statistical results of DMR, CNA and FSR were analysed and the results showed differences between the groups (Figure 3B). A total of 264 DMR regions were identified. The DLBCL cohort showed a decrease in the methylation rate of DMR regions compared to the healthy cohort (Figure 3C,D). Except for intron regions, DMRs were mainly clustered in the promoter region, accounting for approximately 20% (Figure S3A,B). There was a total of 567 5‐Mb bin regions in the FSR, and averaging the FSRs of all patients in each bin region of the two different cohorts revealed that the mean FSR of the DLBCL cohort was higher than that of the healthy cohort in each bin interval (Figure 3E). Meanwhile, the CNA of the DLBCL cohort relative to the healthy cohort was obtained for 1942 1‐Mb intervals, revealing 1283 regions of copy number changes and a total of 686 regions of copy number gain (Figure 3F).

**FIGURE 4:**
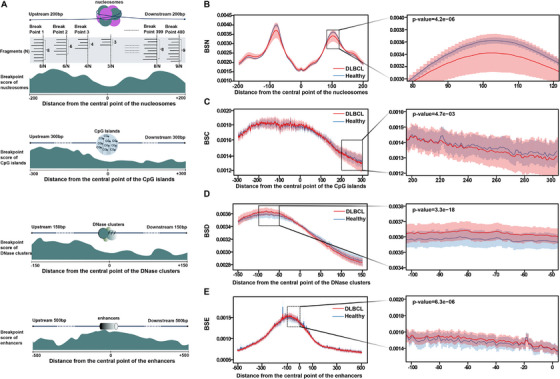
Difference characteristics analysis of BSN, BSC, BSD and BSE. (A) Schematic of breakpoint score of nucleosomes, CpG islands, DNase clusters and enhancers. Setting nucleosomes, CpG islands, DNase clusters and enhancers as the centre point, respectively, counting all cfDNA fragments within 200, 300, 150 and 500 bp upstream and downstream of the centre point, as well as the number of cfDNA fragments at the breakpoint. A high score indicates that there are more cfDNA breakpoints here, and a low score indicates that there are fewer. (B−E) Breakpoint scores of BSN, BSC, BSD and BSE in different ranges between the healthy group and the DLBCL group. The dark red/dark blue line represents the average score of the DLBCL/the healthy group, the light red/light blue area represents the average score ± SD (standard deviation). For the different omics results on the left side of the figure, the area within the dotted box is partially enlarged and displayed on the right side of the figure. Use the rank sum test method to conduct a significance test between the DLBCL group and healthy group in a local area.

Most importantly, the cfDNA breakpoint patterns in regions surrounding chromatin‐associated features, including nucleosomes, CpG islands, DNase clusters and enhancers, were analysed in depth to compare differences between the healthy and cancer groups. To quantify this difference, a breakpoint score was developed. Specifically, using chromatin‐associated features as a centre point, all cfDNA fragments within 200, 300, 150 and 500 bp upstream and downstream of the centre point were counted, as well as the number of cfDNA fragments at the breakpoint. The breakpoint score was calculated as the ratio of the latter to the former (Figure 4A). In certain regions, the differences tended to be present and varied (Figure 4B−E). Taking BSN as an example, the breakpoint score of the DLBCL cohort fluctuated greatly in the area upstream and downstream of the nucleosome‐occupied region compared to the healthy control cohort, showing extremely significant differences between the two groups in one local area. The breakpoint score was lower in nucleosome occupancy regions than that in nucleosome binding regions, consistent with previous studies.^39^ Similarly, there were significant differences in some local areas of BSC, BSD and BSE. In a word, the multi‐omics results produced by COMOS provided comprehensive and robust data underpinning for the construction of early diagnosis models.

### 3.3 COMOS performance in DLBCL early diagnosis

**FIGURE 5:**
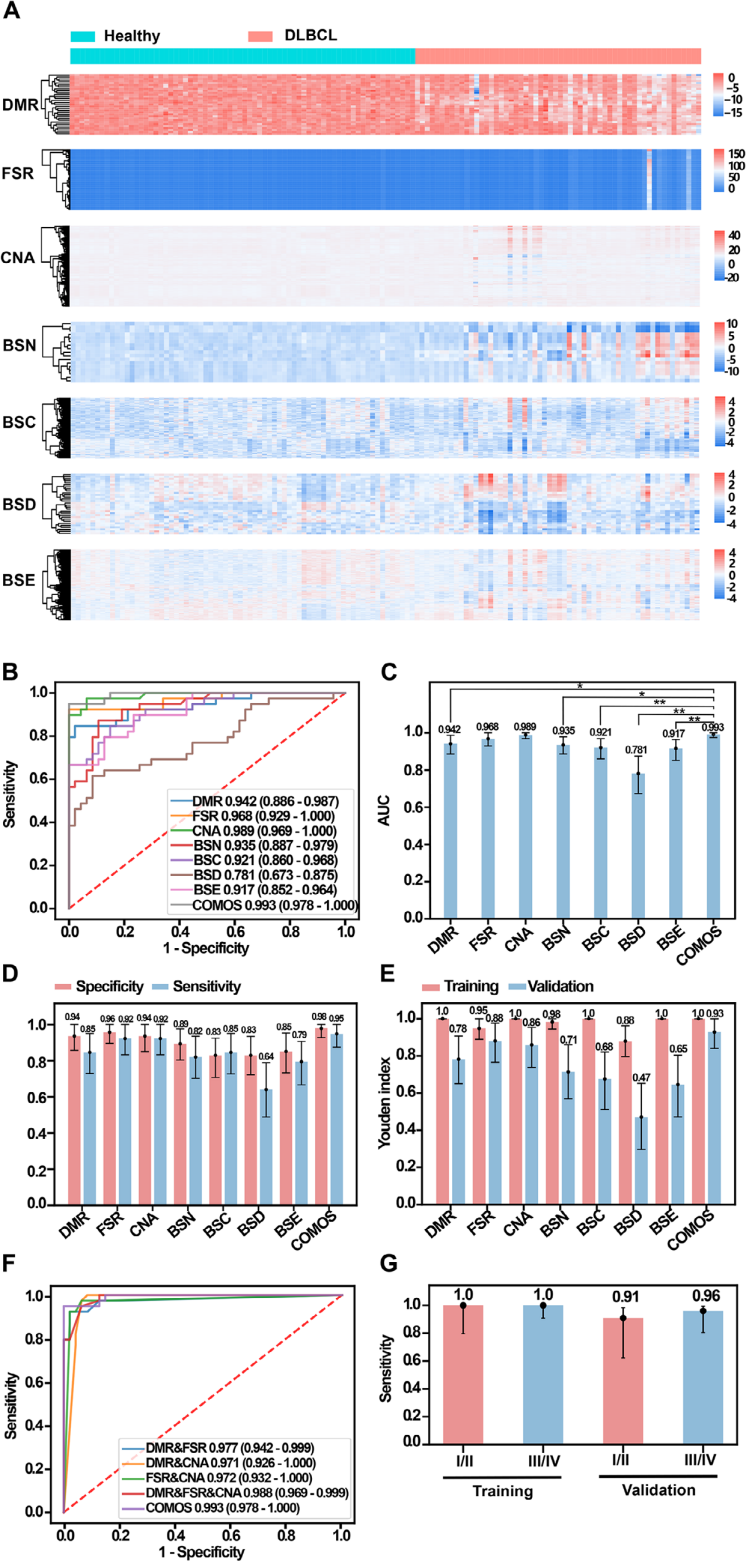
COMOS performance for DLBCL early diagnosis. (A) Difference heat map analysis of cancer patients with different omics characteristics in the training set based on normal controls. (B) Evaluation of the individual omics model and COMOS on the validation set, and calculation of the 95% confidence interval. (C) Performance of the COMOS and the individual omics model on the validation set estimated by DeLongtest. The error bars represent the 95% confidence interval (*p < .05, **p < .01). Only significant differences with COMOS were shown. (D) Specificity and sensitivity of the test set were evaluated using classification thresholds (DMR: .5, FSR: .5, CNA: .5, BSN: .51, BSC: .5, BSD: .55, BSE: .5, COMOS: .5), indicated by error bars 95% confidence interval. (E) Youden evaluation of the individual omics model and COMOS on the training set and validation set, and calculation of the 95% confidence interval. (F) Evaluation of the DMR&FSR, DMR&CNA, FSR&CNA, DMR&FSR&CNA and the COMOS on the validation set, and calculation of the 95% confidence interval. (G) Early diagnostic sensitivity of the COMOS on early‐stages (I and II) and late‐stages (III and IV) at 99% specificity, and 95% Wilson confidence intervals were calculated.

In the training set, through comparative analysis of healthy controls and DLBCL patients, the candidate feature of each group was screened out using RFECV, and a differential heat map was obtained (Figure 5A). The results showed that in addition to FSR and CNA, other omics could distinguish healthy controls from cancer patients to a certain extent. At the same time, the characteristics of all omics through tSNE (Figure S4) were visually analysed, suggesting that DMR could significantly discriminate cancer patients from healthy controls (Figure S4A). Based on the above‐selected omics features, a set of classification models, as well as integration models using the selected DMR, FSR, CNA, BSN, BSC, BSD and BSE were constructed (Table S3) and evaluated on the training and validation sets (Figure 5B, Figure S5A and Table S2). The results showed that COMOS had the highest AUC of .993 (95% CI: .978–1). Further analysis of the model AUC revealed that COMOS was significantly better than DMR (*p* < .05), BSN (*p* < .05), BSC (*p* < .01), BSD (*p* < .01) and BSE (*p* < .01) (Figure 5C). By adjusting the classification threshold on the training set, the sensitivity at 99% specificity of the training set was obtained (Figure S5B). In the validation set, COMOS demonstrated a detection sensitivity of 95% (95% CI: 88−100%) and a specificity of 98% (95% CI: 93−100%) using the same classification threshold (Figure 5D and Figure S5C). COMOS calculated a score corresponding to the probability of being a tumour patient, and the results showed that the healthy control group and the DLBCL group were effectively identified (Figure S5D). As the classification threshold was changed, the sensitivity, specificity and accuracy of COMOS fluctuated in a gradual manner (Figure S5E). In addition, the Youden Index of COMOS on the validation set was .927 (95% CI: .838−1) (Figure 5E), which was superior to that of the individual omics model, suggesting that COMOS had notable advantages in sensitivity and specificity. Furthermore, we randomly combined DMR, FSR and CNA to construct integrating models. The integrating model containing FSR was improved to a certain extent. The AUC value and specificity of COMOS, integrating BSN, BSC, BSD and BSE were superior to the multi‐omics model mentioned above. These results indicated that the fragmentation modalities further exploited the potential of the fragmentomics and improved the performance of COMOS (Figure 5F and Figure S5F).

At the same time, to further evaluate the diagnostic performance of COMOS in different stages of DLBCL, a total of 89 patients with clinical stages were obtained, and the early stage (stages I and II, 11 validation set samples out of 26 samples) and the late stage (III and IV, 25 validation set samples out of 63 samples) were evaluated. In the validation set, the sensitivity for early‐stage and late‐stage DLBCL reached 91% (95% CI: 62−98%) and 96% (95% CI: 80−99%), respectively (Figure 5G). Our findings indicated that COMOS performed much better in the early diagnosis of DLBCL.

### 3.4 Multi‐omics profiling between partial response/complete response and progressive disease/stable disease patients

**FIGURE 6:**
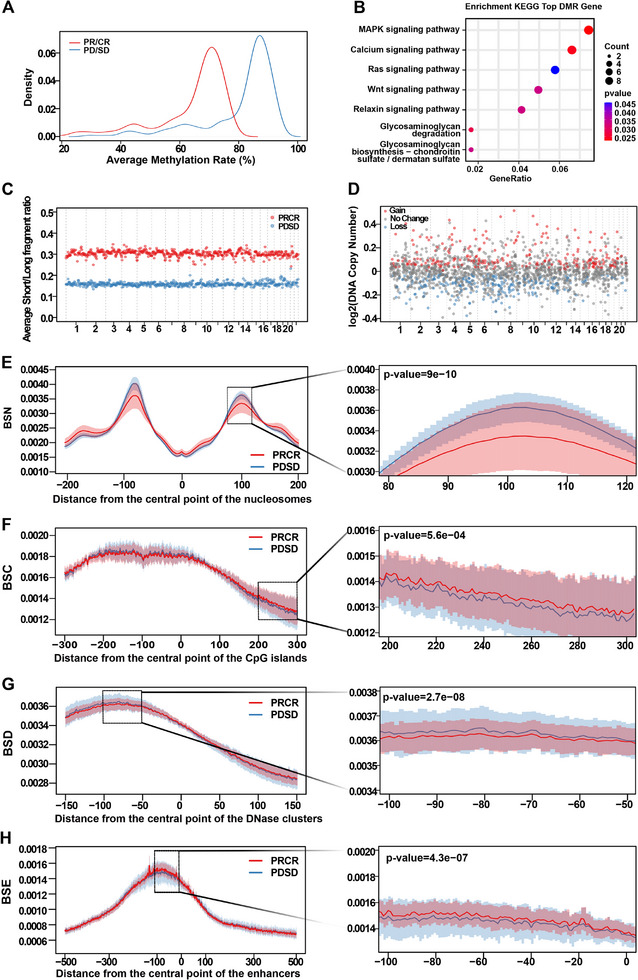
Difference characteristics analysis of methylation, CNA, fragmentation and breakpoint scores in treatment response prediction cohort. (A) Methylation rate density distribution curve in DMR. (B) Partial KEGG pathway enrichment results of genes related to the DMR region. (C) Average FSR of PR/CR group and PD/SD group under each 5 Mb interval. (D) The copy number changes in the PR/CR group relative to the PD/SD control group, that is log2 (PR/CR/PD/SD) and the rank sum test is performed. When *p*‐value 0< 0.05 and log2 (PR/CR/PD/SD) was greater than 0 or less than 0, it was judged as copy number Gain or Loss, otherwise, the copy number was regarded as No Change. (E−H), Breakpoint scores of BSN, BSC, BSD and BSE in different ranges between the PR/CR group and the PD/SD group. The dark red/dark blue line represents the average proportion of the PR/CR group/PD/SD group, the light red/light blue area represents PR/CR group/PD/SD group average score ± SD (standard deviation). For the different omics results on the left side of the figure, the area within the dotted box is partially enlarged and displayed on the right side of the figure. Use the rank sum test method to conduct a significance test between the PR/CR group and PD/SD group in a local area.

According to treatment response, patients were defined as progressive disease (PD), stable disease (SD), partial response (PR) and complete response (CR).^49^ PD and SD were assigned to the R‐CHOP ineffective group, while PR and CR to the R‐CHOP effective group. Besides, 80 patients were divided into a training set and a validation set (Table S4). To explore the potential of COMOS in predicting R‐CHOP treatment response, the statistical results under different omics were analysed. DMR region exhibited lower methylation levels in the PR/CR patient group compared to the PD/SD patient group (Figures 6A and S6A,B). KEGG analysis suggested that DMR‐related genes were enriched in the MAPK signalling pathway, calcium signalling pathway, Ras signalling pathway and other pathways closely related to tumour occurrence and development (Figure 6B). In each bin interval, the average of FSR in PR/CR patient cohort was higher than in the PD/SD patient cohort (Figure 6C). In CNA, the PR/CR patient cohort had 260 gain regions and 231 loss regions compared to the PD/SD patient cohort (Figure 6D). Similarly, the statistical results of the BSN, BSC, BSD and BSE also showed varying degrees of difference (Figure 6E−H). These data demonstrated that COMOS could extract faithful multi‐omics information from the PR/CR and the PD/SD patient cohort.

### 3.5 COMOS performance in DLBCL response prediction

**FIGURE 7:**
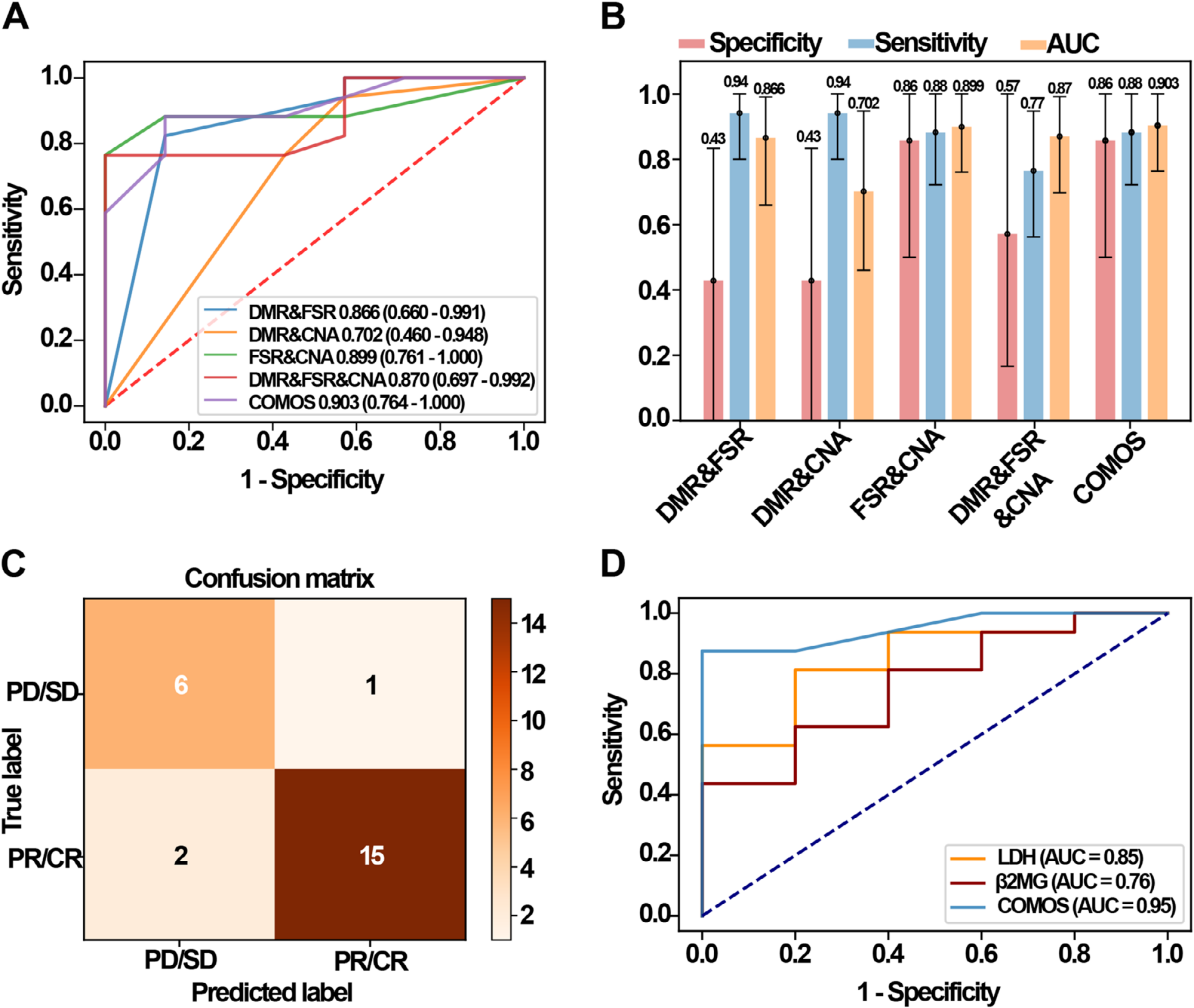
COMOS performance for DLBCL treatment response prediction. (A) Evaluation of the DMR&FSR, DMR&CNA, FSR&CNA, DMR&FSR&CNA and the COMOS on the validation set, and calculation of the 95% confidence interval. (B) The specificity, sensitivity and AUC of the validation set were evaluated using a classification threshold of 0.5, indicated by error bars 95% confidence interval. (C) Confusion matrix of the ensemble COMOS model under validation set evaluation. (D) Comparison of the performance of the COMOS and LDH/β2MG biomarkers in predicting treatment response.

In the training set, through the comparative analysis of PD/SD control and PR/CR groups, the candidate features of each omics were screened by RFECV, and the characteristics of all omics were visually analysed by tSNE (Figure S7). The results showed that the DMR characteristic sites (Figure S7A) could clearly separate PR/CR and PD/SD cohorts, supporting their role in treatment response detection. An integrating multiple classification models on the basis of the above‐selected omics features were constructed (Table S5), and the performance was evaluated on the training and validation sets (Figure S8A,B). The results showed that the AUC of COMOS reached .903 (95% CI: .764–1), which was better than any individual omics model, and the AUC difference analysis showed that the COMOS performance was significantly better than CNA (*p* < .05), BSC (*p* < .01) and BSD (*p* < .05) (Figure S8C). In addition, the performance of COMOS was compared with a randomly combined model of DMR, FSR and CNA. Interestingly, COMOS also achieved the best AUC value (Figure 7A), and the sensitivity at 99% specificity of the training set was obtained by adjusting the classification threshold (Figure S8D). In the validation set, COMOS sensitivity reached 88% (95% CI: 72−100%) and the specificity was 86% (95% CI: 50−100%) (Figure 7B,C and Figure S8E). Although the sensitivity and specificity of COMOS were the same as those of the FSR model and the FSR&CNA model, the AUC value was the highest, indicating that BSN, BSC, BSD and BSE improved the performance of COMOS in predicting the treatment response of DLBCL. The sensitivity, specificity and accuracy of COMOS showed a certain stability when the classification threshold was changed (Figure S8F).

In addition, the predictive performance of the integrating model was also compared with DLBCL prognostic indicators, such as LDH and β2MG (Figure 7D). In the validation set, 21 patients had LDH and β2MG data available (16 PR/CR, 5 PD/SD). The performance of COMOS and the two biomarkers was calculated and compared, showing an AUC of .95 for the integrating model, better than .85 and .76 for LDH and β2MG, respectively. Thus, COMOS achieved a much better predictive performance in comparison to the classical approach by integrating multiple fragmentation features.

## 4 DISCUSSION

In this study, the COMOS was developed to integrate methylation, fragmentation, CNA and four fragmentation modalities of cfDNA, maximizing the application of methylomic information compared with previous studies that only detected limited methylation markers.^50^, ^51^ TWMS could accurately detect different methylation rate values from 0% to 100%, and the DNA sample input could be as low as 1 ng. We have demonstrated that cfDNA CNA and fragmentation analysis can be effectively performed based on the capture data of the entire methylome.

Multi‐omics characteristics of cfDNA can effectively improve the performance of early diagnosis of cancer. However, it was not easy to obtain multi‐omics information in a single reaction using trace amounts of cfDNA. Recently, some studies have used methylation sequencing to simultaneously obtain methylation, fragmentation and copy number variation information, and constructed integrating models to achieve early screening of multiple cancers.^34^, ^52^ However, these studies all obtained common multi‐omics through low‐depth whole‐genome methylation sequencing, and were unable to obtain further omics information. Therefore, the COMOS further explored the omics information of chromatin‐related features in addition to methylation, copy number variation and fragmentation, and obtained a more comprehensive multi‐omics landscape. The COMOS identified four fragmentation modalities, including BSN, BSC, BSD and BSE, leading to a higher AUC value compared to individual omics models and randomly integrating models using DMR, FSR and CNA. COMOS had an AUC value of .993 (95% CI: .978−1) with a sensitivity of 95% and a specificity of 98% for the early diagnosis of DLBCL. In the early‐stage patients, detection sensitivity achieved 91% with a specificity of 99%. Overall, these results suggested that the fragmentation modalities could further extend the benefits of the fragmentomics and contribute to the performance of the model.

R‐CHOP was the gold standard for first‐line treatment of DLBCL, but 30–50% of patients still failed to treatment. Current technical tools for predicting treatment response, such as PET/CT and IPI, all had certain limitations. According to the Guidelines for the Diagnosis and Treatment of Diffuse Large B‐Cell Lymphoma (2022 Edition), the diagnosis of DLBCL mainly relies on clinical manifestations, pathological examinations, imaging examinations, laboratory tests and so on, and these traditional detection methods have certain limitations. For example, pathological examinations require blood pathology experts to evaluate the resected biopsy specimens and perform immunohistochemical analysis on tissue pathological sections to clarify the diagnosis of DLBCL, but specimens must be obtained through surgical resection or coarse needle puncture of lymph nodes or extra‐lymph node tissues, which is invasive to patients. If case specimens are difficult to obtain, it will greatly increase the difficulty of diagnosis. Due to the clinical heterogeneity of DLBCL, traditional diagnostic techniques may obtain less accurate information.^53^ Similarly, in terms of evaluating treatment response, the positive predictive value of PET/CT for the efficacy evaluation of DLBCL is usually very low, mainly due to the inflammatory response after the end of treatment. There is also controversy over the evaluation time of PET/CT. PET/CT at inaccurate time points can also lead to false positive results,^54^ and the cost is also very expensive. The COMOS designed in this study can non‐invasively diagnose DLBCL with only one tube of peripheral blood, without the need for a biopsy specimen, and is patient‐friendly. In early diagnosis, COMOS has a sensitivity of up to 95% and a specificity of 98%. Detection sensitivity achieves 91% at 99% specificity in early‐stage patients. In terms of treatment response prediction, COMOS can predict and evaluate the effectiveness or ineffectiveness of R‐CHOP before treatment, with a superior sensitivity of 88% at 86% specificity (AUC .903), and can provide efficacy results in advance, which greatly provides clinical diagnosis and treatment methods. Compared to the existing treatment response prediction methods using molecular markers such as LDH and β2MG, the AUC values of LDH and β2MG are .85 and .76, respectively, and COMOS had the highest detection accuracy. In summary, COMOS has great advantages in diagnosis and treatment response prediction research, such as timeliness, sampling convenience, friendliness and high accuracy.

It is known that DLBCL has different genetic polymorphisms, which are associated with ethnicity and geography. The cohort we studied covered DLBCL patients in northern and southern China, including Han and ethnic minorities from different regions, and we obtained multiple omics information on methylation, fragmentation, CNA and chromatin‐related characteristics, providing a wide range of information for populations of different ethnicities and geographies. We used this broader, inclusive information to build early diagnosis and treatment response prediction models, which significantly enhanced the generalizability of the models across diverse ethnic and geographic populations with DLBCL. However, our cohort did not include people from Europe and the United States, so our study has certain limitations for DLBCL patients in Europe and the United States.

In addition to robust performance, another key advantage of COMOS is flexibility. COMOS can extract seven omics information simultaneously, providing rich features for integrating models. Although this study was developed for DLBCL, the inclusion or exclusion of specific omics information was adjustable when applied to different cancer types. In addition, all omics information was captured simultaneously in a single workflow, which improved detection efficiency and reduced costs. Overall, COMOS had great advantages for cfDNA samples with low amount. Seven omics information can be obtained in a single reaction, providing a more comprehensive and flexible combination of molecular markers for model integration.

Although COMOS has made some breakthroughs in the early diagnosis of cancer and the prediction of treatment response scenarios, this research still has some limitations. First, in the early detection study, due to the small number of stage I samples, we collectively referred to stage I and stage II patients as early stage and stage III and stage IV patients as late stage. Although COMOS showed high sensitivity for early‐stage cancer, the limited sample number in the early cohort required further evaluation. Second, the confidence interval varied widely in the treatment response prediction mode, which may be due to the small number of samples received or the difficulty in assessing the treatment response endpoint. Third, as this is a retrospective case‐control study, an accurate assessment of the real‐world performance of COMOS in a larger prospectively screened cohort with complete long‐term follow‐up is required.

## 5 CONCLUSIONS

In summary, we have developed COMOS, a comprehensive and integrative approach with inspiring sensitivity and specificity in the early diagnosis of DLBCL and the prognosis of R‐CHOP treatment. As current sequencing techniques offer only small pieces of fragmentomics, the COMOS enables the identification of four innovative metrics, including BSN, BSC, BSD and BSE, as well as the classic DMR, CNA and FSR. This integrative strategy not only provides a more complete and detailed fragmentation landscape of the molecular information contained in cfDNA, but also represents a revolutionary advancement in the diagnostic and prognostic capabilities of liquid biopsies, holding significant potential for early diagnosis and treatment response prediction in various clinical applications.

## AUTHOR CONTRIBUTIONS

BY, ZZ and WZ: Conceived the project; WZ, BY and YS: Developed and designed the methodology; BY, YS and HJ: Analysed and synthesized study data; YS and DY: Created the models; BY, FY and YS: Validated the overall experiments; WZ, FY, PY and WS: Performed the experiments and collected data; WZ, WS, YL and HJ: Contributed patient's blood sample; WZ, HJ, ZZ and YL: Supervised the subject; BY, YS, HJ, CW and ZW: Wrote the initial draft; ZZ, WZ and YL: Reviewed and edited the final draft; YL: Acquired the funding.

## CONFLICT OF INTEREST STATEMENT

Bangquan Ye, Yang Song, Hairong Jing, Fan Yang, Dan Yuan, Zhihong Wu, Jiahao Lyu, Kang Peng, Zijian Zhao and Yanzhao Li are employees of BOE Technology Group Co., Ltd.

## ETHICS APPROVAL AND CONSENT TO PARTICIPATE

The study was approved by the ethic committee of the Peking University Third Hospital. This study was consistent with the principles of the Declaration of Helsinki, and every participant had signed patient consent.

## Supporting information



Supporting Information

Supporting Information

## Data Availability

All data generated or analysed during this study are included in this published article (and its Supplementary Information files). We provided the original dataset (Dataset.NCvsT.txt and Dataset.PDSDvsPRCR.txt) from the early diagnosis and treatment response prediction model, and the features have not undergone any filter steps.
