# Supplementary material for: Integrating multi‐omics features enables non‐invasive early diagnosis and treatment response prediction of diffuse large B‐cell lymphoma
Source: Clin Transl Med. 2025 Jan 7;15(1):e70174. doi: 10.1002/ctm2.70174 (PMC11705727; doi:10.1002/ctm2.70174)

Supplementary file 1 for

**Integrating multi-omics features enables non-invasive early diagnosis and treatment response prediction of DLBCL**

Weilong Zhang, Bangquan Ye, Yang Song, Ping Yang, Wenzhe Si, *et al.*

**Author information**

Weilong Zhang, zhangwl2012@126.com

Bangquan Ye, yebangquan@boe.com.cn

Yang Song, songyang-b7@boe.com.cn

Ping Yang, yangping198302@163.com

Wenzhe Si, wenzhesi@bjmu.edu.cn

**Supplementary Text**

Fig. S1. Workflow of TWMS. (A), End repair and addition of 'A' bases are performed, cfDNA is ligated with the fully cytosine-methylated UMI adapter, followed by enzyme-mediated methylation transformation, pre-amplification, hybridization with the methylome probe panel, purification and post-amplification. (B), The structure of the fully cytosine-methylated UMI adaptor, which contains three random bases as shown in red color, the Illumina Read1 and Read 2 primer sequences as shown in green color, and all cytosines included in the adapter are methylated. (C), The number of unique reads is compared and analyzed, and the UMI parameters are used or not when removing duplicates. +: UMI parameters are used for sequencing data deduplication analysis, －: UMI parameters are not used for analysis.


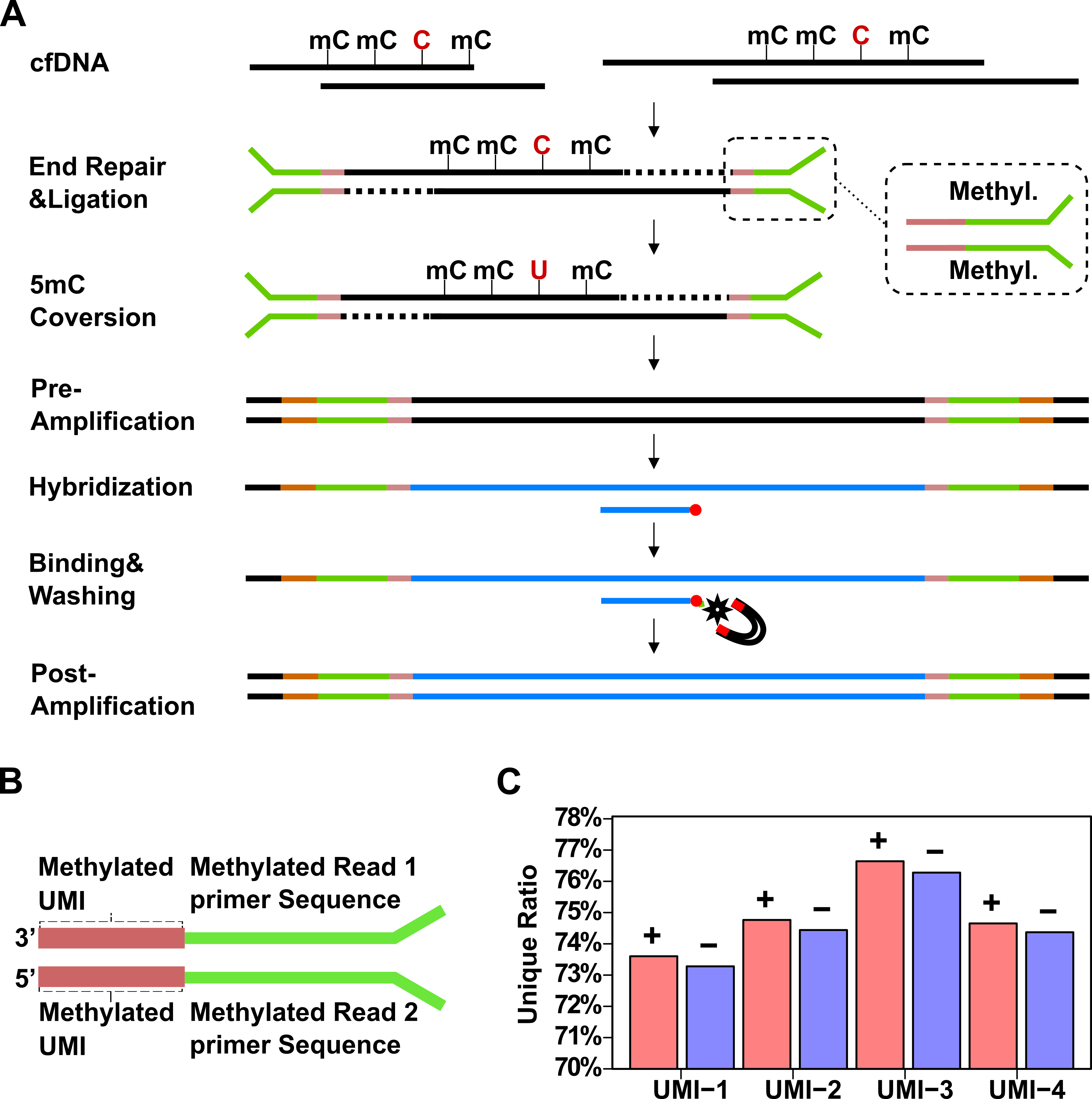


Fig. S2. Concordance between TWMS and Non-TWMS in CNA and fragmentation profiling. (A-C), Scatter plot of CNA profiles from TWMS and non-TWMS data of samples 4-06, 4-65, and 4-36, correlation coefficient (R) of 0.82, 0.85, 0.82 and the p value <0.01 are indicated on the plot. (D-F), Scatter plot of fragmentation profiles from TWMS and non-TWMS data of samples 4-06, 4-65, and 4-36, correlation coefficient (R) of 0.88, 0.92, 0.86 and the p value <0.01.


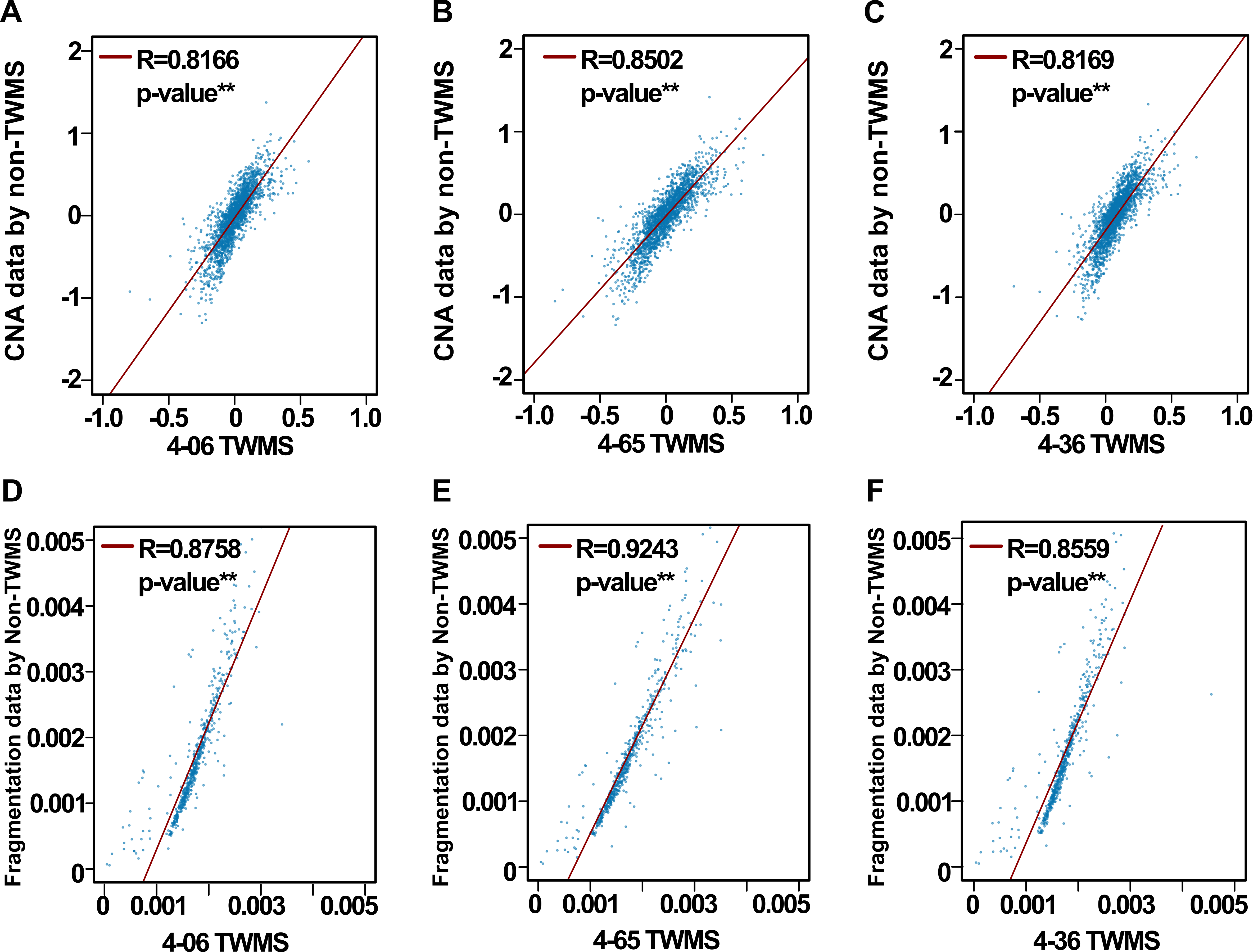


Fig. S3. Distribution and proportion of DMRs in the early diagnosis cohort in the genome. (A), Scatter plot of the distribution of DMR annotations to different original parts of the genome, where one point represents one DMR. (B), Statistics on the number of DMRs annotated to different genomic elements.


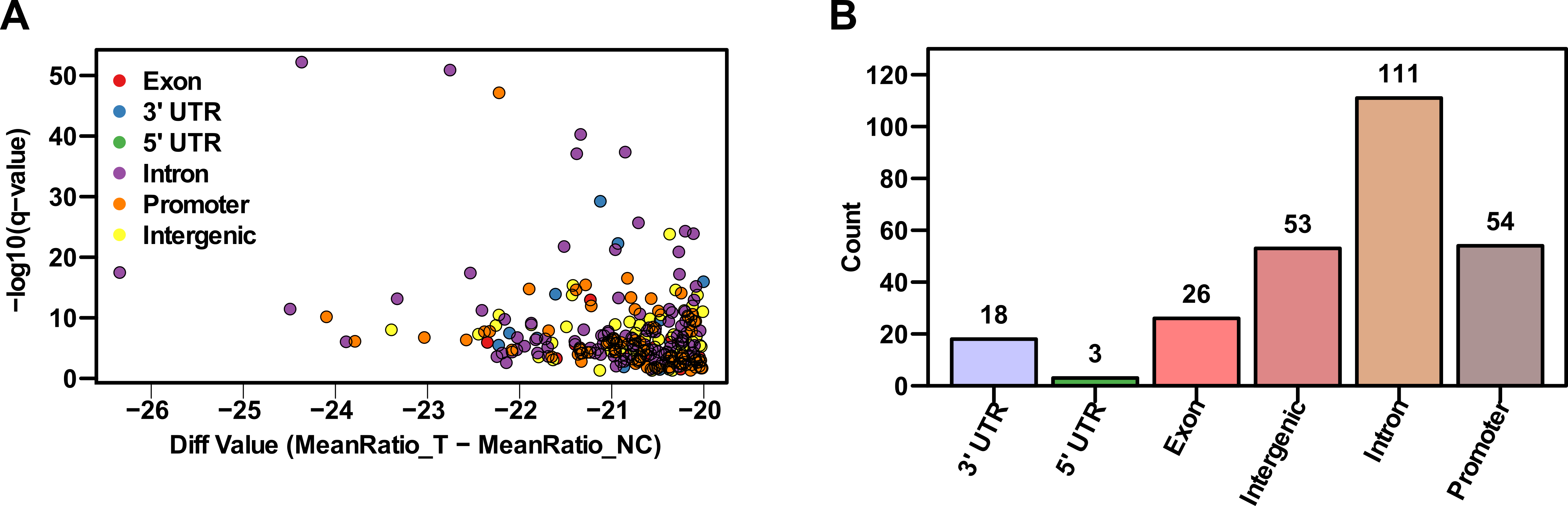


**Fig. S4. Visual analysis of individual omics data by tSNE in early diagnosis cohort.** (**A-G**), tSNE analysis of DMR, FSR, CNA, BSN, BSC, BSD, and BSE featured by RFECV in the training set.


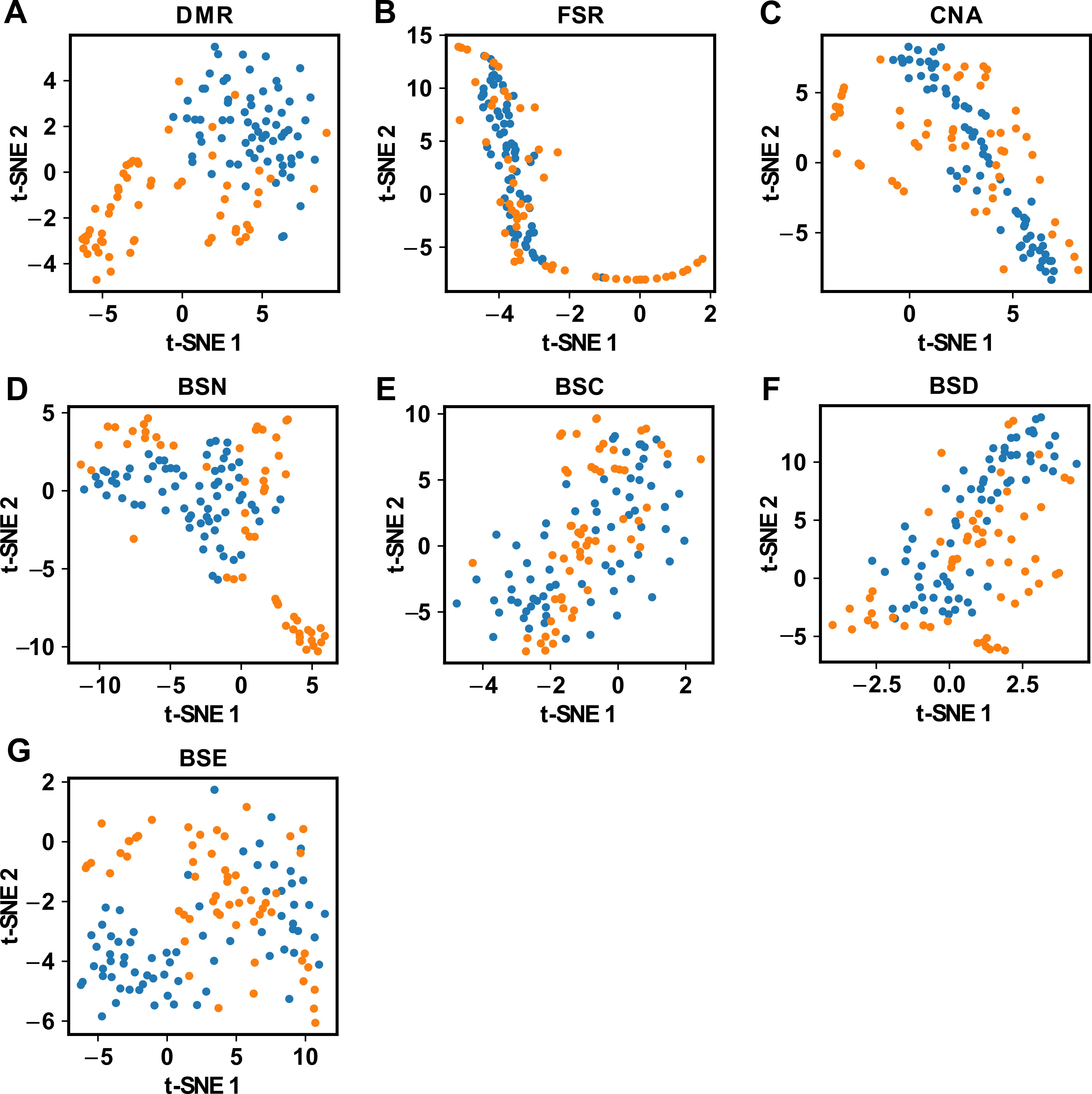


**Fig. S5. Performance evaluation of individual omics and COMOS models in early diagnosis cohorts.** (**A**), Evaluation results of the individual omics model and COMOS in the training set, and calculation of the 95% confidence interval. (**B**), By adjusting the classification threshold on the training set, the sensitivity at 99% specificity of the training set is obtained, and the 95% confidence interval is marked by the error bar. (**C**), Confusion matrix of the COMOS model under validation set evaluation. (**D**), COMOS model score of the healthy and DLBCL groups. (**E**), Changes in the sensitivity, specificity, and accuracy of the COMOS model under different classification thresholds. (**F**), The specificity, sensitivity and AUC of the validation set were evaluated using a classification threshold of 0.5, indicated by error bars 95% confidence interval.


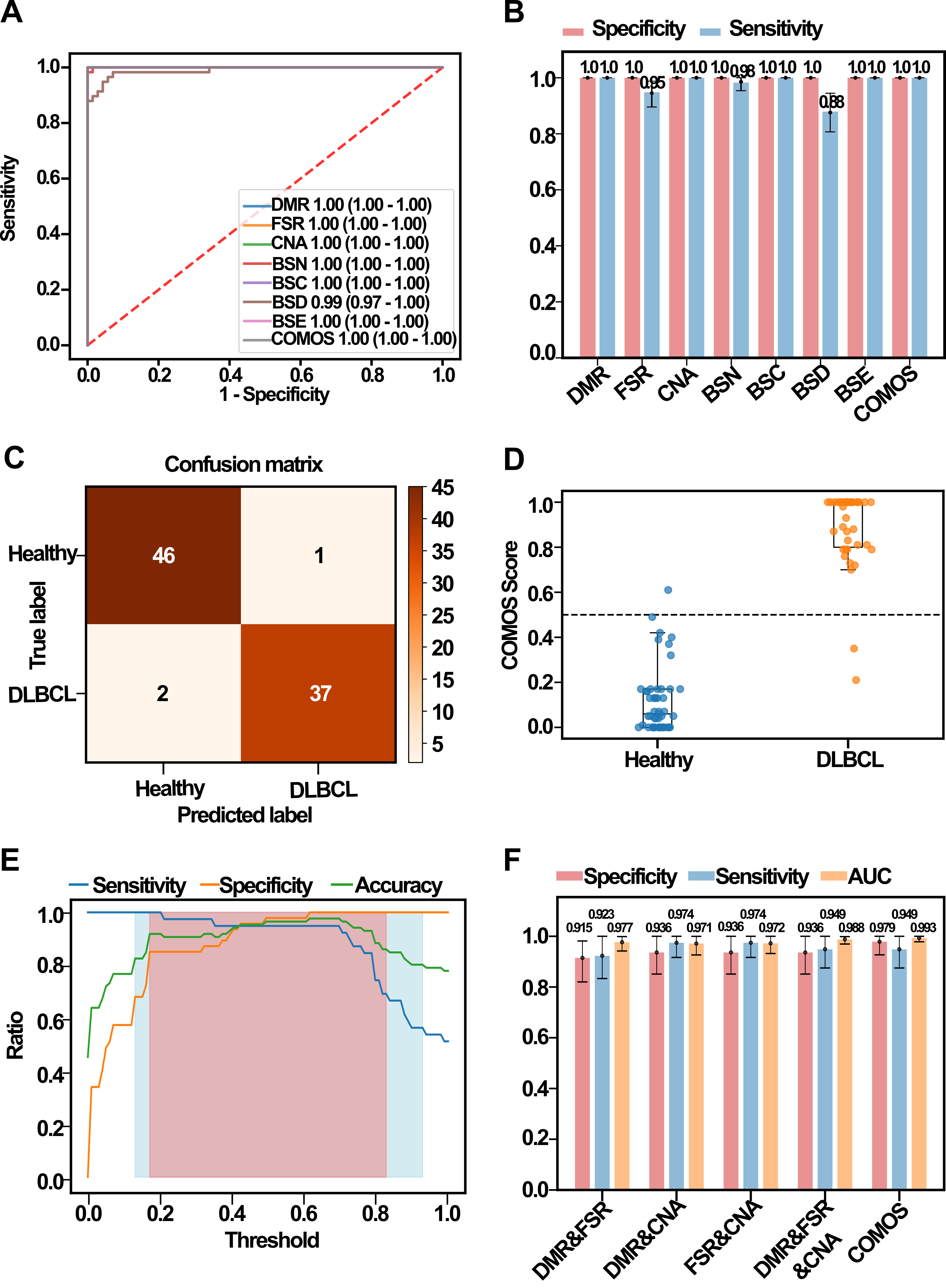


**Fig. S6. Distribution and proportion of DMRs in the treatment response cohort in the genome.** (**A**), Scatter plot of the distribution of DMR annotations to different original parts of the genome, where one point represents a DMR. (**B**), Methylation ratio of the genomic element regions of the PR/CR group and the PD/SD group.


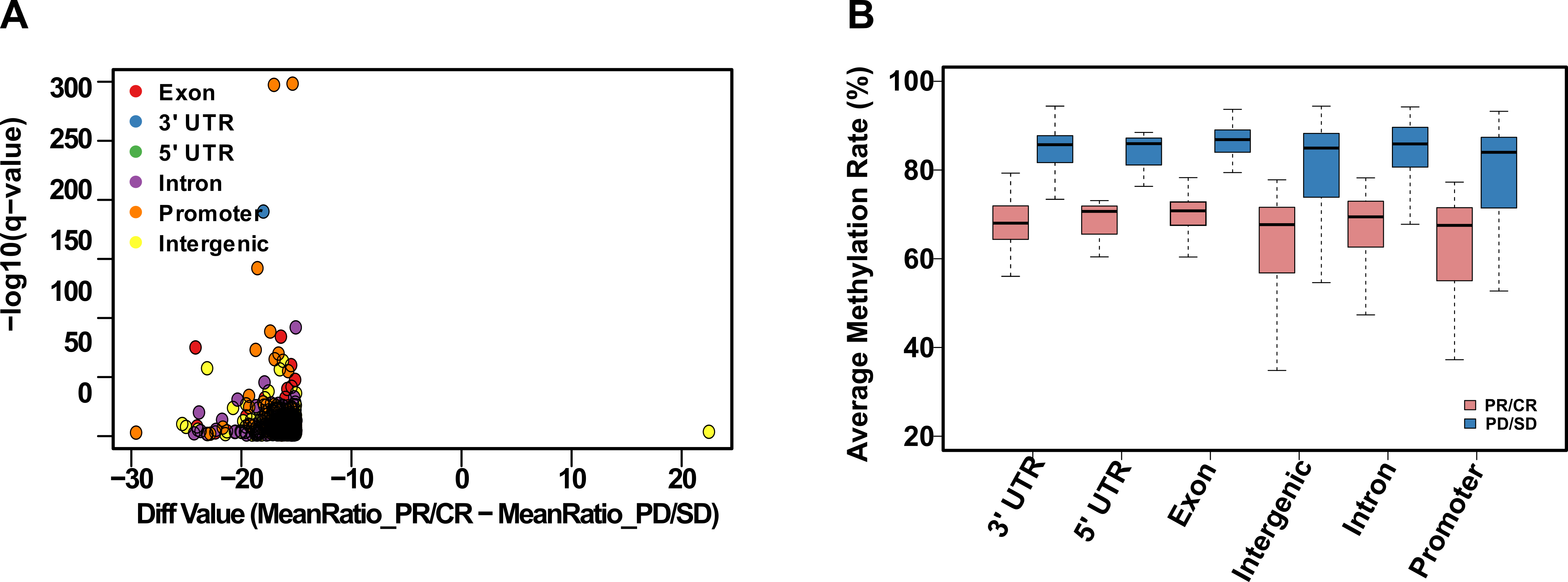


**Fig. S7. Visual analysis of individual omics data by tSNE in treatment response cohort**. (**A-G**), tSNE analysis of DMR, FSR, CNA, BSN, BSC, BSD, and BSE features by RFECV in the training set.


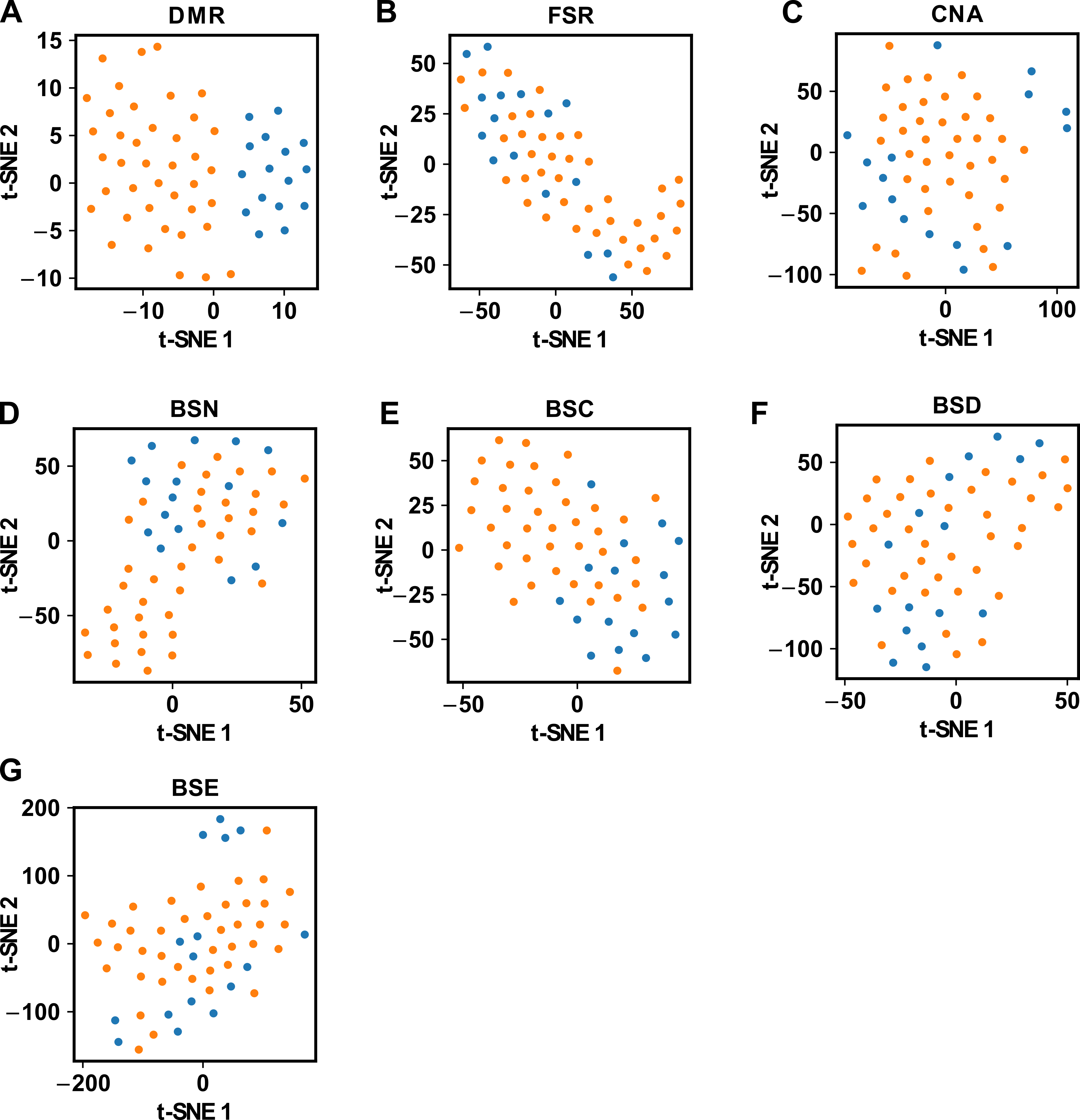


**Fig. S8. Performance evaluation of individual omics and COMOS models in treatment response cohorts.** (**A**), Evaluation results of the individual omics model and the COMOS model on the training set and calculation of the 95% confidence interval. (**B**), Evaluation of the individual omics model and the COMOS on the validation set, and calculation of the 95% confidence interval. (**C**), Performance of the COMOS model and the individual omics model on the validation set estimated by the DeLong test. Error bars represent the 95% confidence interval (*p<0.05, **p<0.01). Only significant differences with COMOS are shown. (**D**), By adjusting the classification threshold on the training set, the sensitivity at 99% specificity of the training set is obtained, and the 95% confidence interval is marked by the error bar. (**E**), The specificity, sensitivity and AUC of the validation set were evaluated using classification thresholds (DMR: 0.69, FSR: 0.5, CNA: 0.5, BTN: 0.5, BTC: 0.84, BTD: 0.5, BTE: 0.5, COMOS: 0.5), indicated by error bars 95% confidence interval. (**F**), Changes in sensitivity, specificity and accuracy of the COMOS model under different classification thresholds.


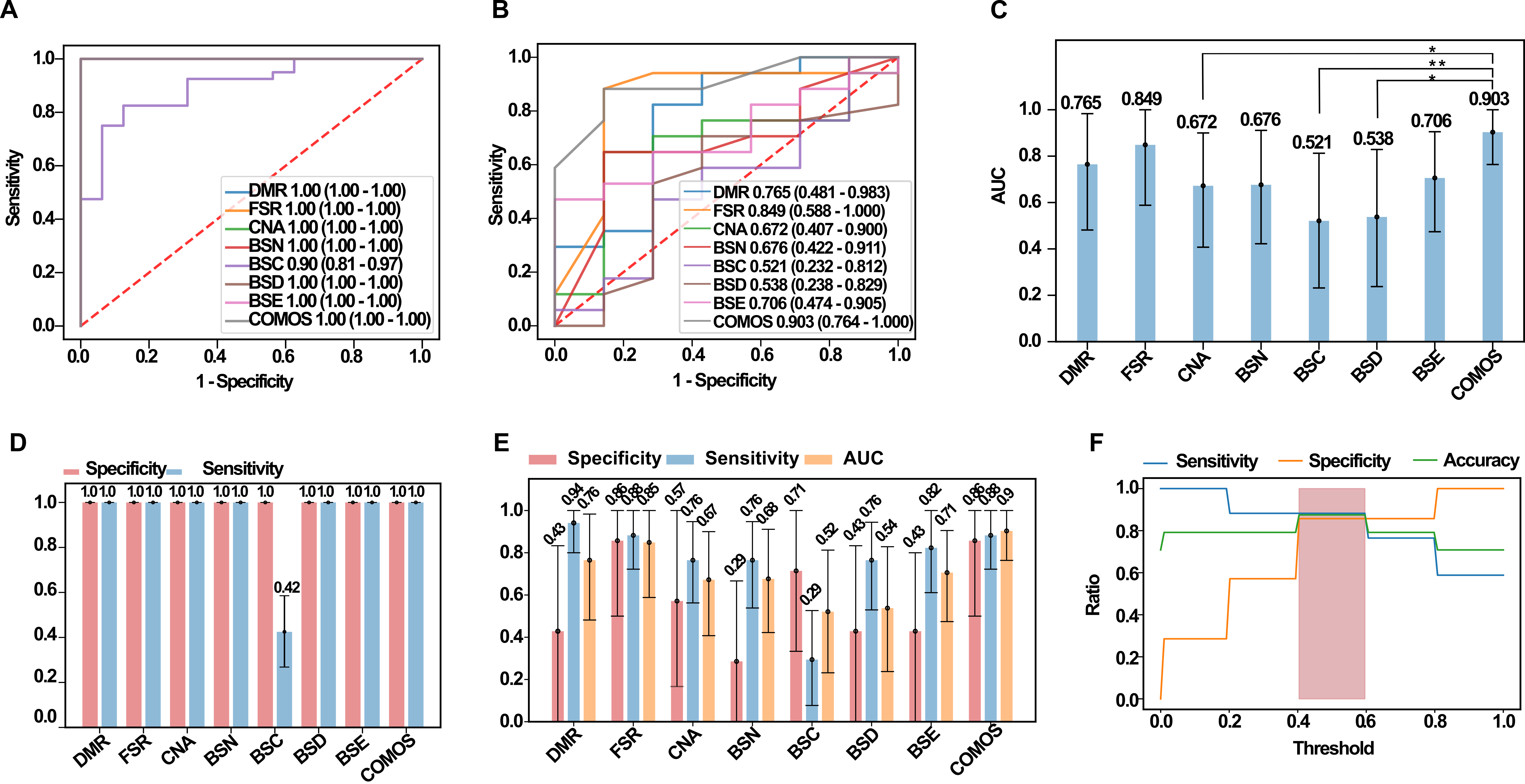

Supplement: Supplementary file 1 — Supporting Information [file CTM2-15-e70174-s002.docx]
